# Influences of Microbial Symbionts on Chemoreception of Their Insect Hosts

**DOI:** 10.3390/insects14070638

**Published:** 2023-07-14

**Authors:** Zhengyan Wang, Zhenzhen Chang, Zhiyuan Liu, Shan Zhang

**Affiliations:** School of Food and Strategic Reserves, Henan University of Technology, Zhengzhou 450001, China; 2021920132@stu.haut.edu.cn (Z.C.); hngydx_lzy@stu.haut.edu.cn (Z.L.); 2022920144@stu.haut.edu.cn (S.Z.)

**Keywords:** insect, microbial symbiont, chemoreception, co-evolution

## Abstract

**Simple Summary:**

Microbial symbionts could intervene with the physiological processes of insect chemoreception, such as changing the processing, transmission, and integration of olfactory or gustatory signals and thereby improve or reduce insect sensitivity to semiochemicals, which in turn adjusts insect behavior. The influence of microbial symbionts on insect chemical communication facilitates the spread of symbionts but could be beneficial or detrimental to insects, which provides the impetus for the co-evolution of symbiotic systems. The association between microbial symbionts and insect chemoreception could be exploited to develop new insect control strategies.

**Abstract:**

Chemical communication is widespread among insects and exploited to adjust their behavior, such as food and habitat seeking and preferences, recruitment, defense, and mate attraction. Recently, many studies have revealed that microbial symbionts could regulate host chemical communication by affecting the synthesis and perception of insect semiochemicals. In this paper, we review recent studies of the influence of microbial symbionts on insect chemoreception. Microbial symbionts may influence insect sensitivity to semiochemicals by regulating the synthesis of odorant-binding proteins or chemosensory proteins and olfactory or gustatory receptors and regulating host neurotransmission, thereby adjusting insect behavior. The manipulation of insect chemosensory behavior by microbial symbionts is conducive to their proliferation and dispersal and provides the impetus for insects to change their feeding habits and aggregation and dispersal behavior, which contributes to population differentiation in insects. Future research is necessary to reveal the material and information exchange between both partners to improve our comprehension of the evolution of chemoreception in insects. Manipulating insect chemoreception physiology by inoculating them with microbes could be utilized as a potential approach to managing insect populations.

## 1. Introduction

Chemical communication is widespread among insects and exploited to adjust their behavior, such as food and habitat seeking and preferences, recruitment, defense, and mate attraction [1]. During the communication process, semiochemicals, such as pheromones and allelochemicals, may be released into the water or air, transported by currents, adhered to food or plants, or transferred through direct contact, e.g., trophallaxis [2], and eventually recognized by insect chemoreceptors, thereby triggering attractive or repellent behavior in insects. Chemoreception, an essential process in the perception and analysis of chemical information from the environment [3], is described as the physiological response to a chemical signal. Chemoreception is divided into olfaction and gustation based on the spatial scale. Olfaction is responsible for detecting volatile molecules originating from afar, while gustation serves as a contact sense that detects molecules with low volatility [4].

Recently, mounting evidence suggests that microbial symbionts play essential roles in insect chemical communication. Many of these roles are usually regulated by microbe-produced chemical signals [5,6]. For instance, the honeydew secreted by aphids involves volatile compounds produced by gut symbionts, which may attract natural enemies and potential ant partners [7]. It has also been found that symbionts, especially pathogens, could affect host chemoperception and improve or reduce host sensitivity to semiochemicals. For example, *Dengue virus* (DENV) infection increases the gene expression in the mosquito’s head and leads to a reduction in olfactory preferences, potentially altering the preference of oviposition sites in female mosquitoes [8]. However, the molecular basis for the association between symbionts and insect chemoreception has not been elaborately revealed in existing studies.

Throughout their long-time evolution, insects have developed close associations with microbial symbionts, and both partners interact with and depend on each other while co-evolving simultaneously [9]. Thus, a deep understanding of the influence of microbial symbionts on insect chemoreception can provide directions and ideas for research on the co-evolution of insect-microbe symbiosis as well as a sound foundation for developing new insect control strategies and species conservation strategies [10,11]. In this paper, we review recent studies of the influence of microbial symbionts on insect chemoreception and their molecular mechanisms and analyze their evolutionary significance, aiming to provide insight into the further study of symbiont-mediated chemoreception in their hosts.

## 2. Physiological Processes of Insect Chemoreception

### 2.1. Insect Olfaction

Insect olfaction is a complex process in which the peripheral olfactory nervous system transforms volatile molecules into electrophysiological signals, which are transported to primary olfactory centers and then integrated and processed in higher nerve centers, ultimately eliciting corresponding behavioral responses in insects [9]. The sensilla on the maxillary palps and antennae are the most crucial olfactory tissues, enabling the insect to detect volatile chemicals [12]. In olfactory sensilla, odor molecules enter the sensilla lymph via pores in the sensilla wall where they bind to soluble odorant-binding proteins (OBPs) or chemosensory proteins (CSPs) [13]. These proteins transfer hydrophobic odorants to membrane-bound olfactory receptors (ORs) or ionotropic receptors (IRs) situated on the dendritic membrane of olfactory neurons through the sensilla lymph. Subsequently, ORs or IRs transform chemical signals into electrophysiological signals and transmit them to the central nervous system (CNS), thereby eliciting an olfactory response [14]. Finally, odor molecules are decomposed by odorant-degrading enzymes (ODEs) to avoid overstimulating olfactory neurons [15].

### 2.2. Insect Gustation

Insects possess a sophisticated gustatory system. The main gustatory organs known as gustatory sensilla are predominantly located on their legs and wings. Each sensillum contains multiple gustatory receptor neurons (GRNs) that express gustatory receptors (GRs), the pickpocket, IRs, or transient receptor potential receptors in their dendrites [16]. The GR genes can be classified into two groups. The first group involves most GR genes (about 35), which are expressed in neurons that detect bitter and salty tastes. The other group includes eight genes only expressed in neurons that are sensitive to the sweet taste [3]. Taste compounds, including amino acids, nucleotides, simple sugars, acids, mineral salts, and various secondary allelochemicals, are the primary ligands of GRs [17]. Unlike the olfactory sensilla, which have many small pores in the sensilla wall, the gustatory sensilla only have a small pore at the tip of the sensilla. Chemicals enter the gustatory sensilla through the pore to contact the dendrites of the GRN. The GRs on the dendrites are activated by chemical molecules to generate electrophysiological signals, which are then transported to the insect CNS in the form of pulses through the axons, thereby regulating the behavior of the insect [18].

## 3. Influence of Microbial Symbionts on Insect Chemoreception

The association between symbionts and insect chemoreception is mostly found in the symbiosis of insects with endosymbionts or pathogens (Table 1). Some microbial symbionts can reduce insect olfactory sensitivity. In the parasitoid wasp *Nasonia giraulti*, the male-to-female transfer of the endosymbiont *Wolbachia pipientis* to uninfected females increases the load of *W*. *pipientis* in the brain or sensory organs of the head region of females, which leads to the increased acceptance of females to interspecific mates [19]. In the Western honeybee *Apis mellifera*, *deformed wing virus* (DWV) infection in the antennae reduces the olfactory sensitivity of middle- and forager-age honeybees to the odor of the essential oils of *Eucalyptus globulus* and *Mentha piperita*, and the increment in the viral load is negatively correlated with the olfaction of honeybees [20]. Exposure to entomopathogenic fungi *Beauveria bassiana* or *Metarhizium acridum* reduces host-seeking responses and olfactory neuron responsiveness tuned specifically to 1-octen-3-ol in the Asian malaria mosquito *Anopheles stephensi* [21].

As the insect host needs energy partitioning between immune reaction and normal physiological activity [22], extremely high loads of endosymbiont or pathogen infections could induce strong energy-consuming immune reactions [23], which may lead to decreased energy input in other processes, including host chemoreception. Therefore, the inhibitory effect of symbionts on insect chemoreception is not necessarily due to the regulation of olfactory physiology by symbionts but may be the result of the trade-off between the immune reaction and investment in host chemoreception. However, some symbionts can enhance the sensitivity of insect chemoreception. In the fruit fly *Drosophila melanogaster*, larvae fed with antibiotics have a lower gut microbial load and exhibit a decreased chemotaxis reaction toward odorants, and this phenomenon is partially rescued in larvae treated with probiotics that results in the partial recovery of the microbial load [24]. This indicates that the microbial load is positively correlated with host chemotaxis response toward odorants. In another study, infection with the endosymbiotic bacterium *Wolbachia* could enhance host olfactory responsiveness, and *Wolbachia*-infected *Drosophila simulans* is more sensitive to ethyl acetate odor than uninfected controls [25]. Similarly, the whitefly *Bemisia tabaci* prefers to feed on healthy plants after the acquisition of *Tomato chlorosis virus* (ToCV), while healthy whiteflies have a feeding preference for ToCV-infected plants [26]. These findings provide direct evidence that microbial symbionts could positively influence host chemoreception.

The association between microbial symbionts and insect gustatory perception is rarely mentioned in existing studies. By comparing the proboscis extension response of germ-free honeybees and normal honeybees, it was found that the gut microbiota significantly elevates host sucrose sensitivity, and normal honeybees respond to lower concentrations of sucrose [27]. However, infection of the tiger moth, *Grammia geneura*, with lethal parasites results in the altered taste perception of specific phytochemicals in the caterpillars. Compared with unparasitized caterpillars, the taste cells of parasitized individuals increase their firing rates when exposed to pyrrolizidine alkaloids and iridoid glycosides that are toxic to parasitoids but reduce their firing rates in response to deterrent caffeine, which drives the caterpillar to ingest normally unpalatable plants. It was also found that there are no differences in the response of caterpillars to sucrose, a phagostimulatory nutrient. As the sucrose sensitivity is mediated by the same gustatory neuron as iridoid sensitivity, it implies that the modification of sensory neurons does not influence the whole cell but rather certain receptor proteins or their second messengers [28]. It also suggests that the parasite-associated taste change should be an observed phenotype of self-medication, so it is rather a host-controlled change than a parasite-controlled one.

**Table 1:** Influences of microbial symbionts on insect chemoreception.

Insect Hosts	Presence of Microbial Symbionts	Influences of Symbionts on Insect Hosts
*Aedes aegypti*	*Dengue virus* [Virus]	Upregulate AeObp22 expression [29]
	Double subgenomic *Sindbis virus* [Virus]	Downregulate expression of *AaegObp1* and *AaegObp2* [30]
	*Wolbachia* [Bacterium]	Increase dopamine levels [31]
*Apis mellifera*	*Deformed wing virus* [Virus]	Downregulate OBP expression [20]
	Gut microbiota [Bacterium]	Elevate olfactory sensitivity to sucrose [27]
	Gut *Lactobacillus* [Bacterium]	Improve learning and memory performance [32]
	*Nosema ceranae* [Microsporid]	Downregulate OBP expression [33]
*Bemisia tabaci*	*Tomato chlorosis virus* [Virus]	Upregulate *OBP3* expression [26]
*Caenorhabditis elegans*	*Providencia* [Bacterium]	Modulate an aversive olfactory response [34]
*Drosophila melanogaster*	*Erwinia carotovora carotovora 15* [Bacterium]	Decrease olfactory discrimination [35]
	Gut microbiota [Bacterium]	Increase chemotaxis response to odorants [24]
	Gut microbiota [Bacterium]	Reduce memory of olfactory appetitive [36]
	*Wolbachia* [Bacterium]	Upregulate expression of *Pale* and *Ddc* [37]
*Drosophila simulans*	*Wolbachia* [Bacterium]	Upregulate expression of *or83b* [25]
*Glossina morsitans morsitans*	*Wigglesworthia* [Bacterium]	Upregulate expression of *obp6* [38]
*Locusta migratoria*	*Metarhizium anisopliae* [Fungus]	Upregulate expression of *LmOBP11* [39]
*Nasonia giraulti*	*Wolbachia pipientis* [Bacterium]	Increase mate acceptance of infected females [19]
*Solenopsis invicta*	*Beauveria bassiana* [Fungus]	Upregulate expression of *SiCSPs* and *SiOBPs* [40]
*Spodoptera exigua*	*Spodoptera exigua multiple nucleopolyhedrovirus* [Virus]	Upregulate *OR35* expression [41]
*Trichogramma brassicae*	*Wolbachia* [Bacterium]	Decrease memory retention [42]

## 4. Mechanism of Symbiont-Mediated Insect Chemoreception

**Figure 1:**
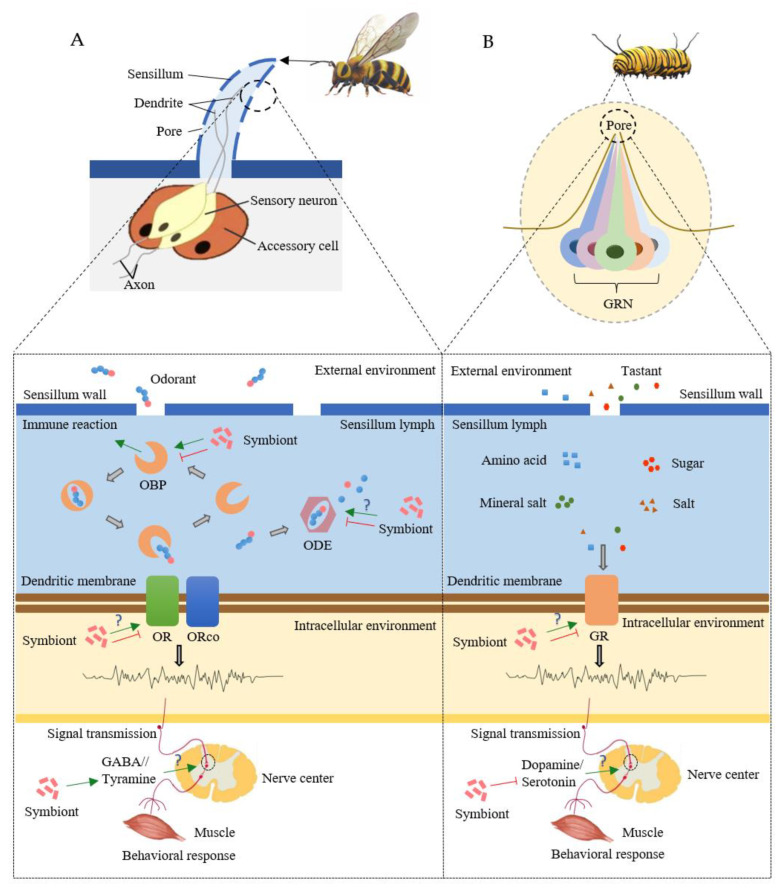
Symbiont-mediated physiological processes of insect olfaction (**A**) and gustation (**B**). Green arrows represent stimulatory effects and red arrows represent inhibitory effects. GABA, gamma-aminobutyric acid; GR, gustatory receptor; GRN, gustatory receptor neuron; OBP, odorant-binding protein; ODE, odorant-degrading enzyme; OR, olfactory receptor; ORco, odor receptor co-receptor.

Microbial symbionts could intervene with the physiological processes of insect chemoreception, such as changing the processing, transmission, and integration of olfactory or gustatory signals [9] (Figure 1).

### 4.1. Regulating Host OBP/CSP Expression

Microbial symbionts, such as viruses, gut bacteria, and entomopathogenic fungi, can enhance insect sensitivity to chemical signals by regulating the expression of OBPs. DENV infection leads to an increased AeOBP22 expression within the mosquito salivary gland [29], and ToCV infection stimulates *OBP3* expression in whiteflies [26]. Obp14 expression in *A*. *mellifera* is upregulated by gut *Lactobacillus* Firm4 and Firm5 [43]. Infection with the pathogenic fungus *B*. *bassiana* stimulates the expression of *SiCSPs* and *SiOBPs* in the fire ant *Solenopsis invicta* [40]. Obviously, the overexpression of OBPs/CSPs can mediate the host detection of pathogens and contribute to subsequent behavior, such as grooming and nest sanitation in ants to mitigate the dispersal of microbial pathogens within their communities [44].

OBPs share structural similarities with other proteins involved in the binding and transportation of other signals, such as RYA3, that displays critical sequence homology to the lipopolysaccharide (LPS)-binding protein [45]. This raises the probability that OBPs function as a main defense mechanism by recognizing and/or neutralizing invading microbes. The pores of chemosensory sensilla provide an easy entry for microbes, and mechanisms to keep sterility of the sensilla lymph are likely to exist, possibly involving the expression of OBPs. The fact that the antenno-maxillary complex, which regulates larval olfaction, produces two antimicrobial peptides when exposed to bacteria supports the presence of host-defense mechanisms related to olfactory organs in *Drosophila* larvae [46]. Pherokines, including Phk-1, Phk-2, and Phk-3, tissue-specifically expressed in the olfactory organs and structurally and empirically characterized as putative OBPs are upregulated after the infection of *D. melanogaster* with the picorna-like *Drosophila C virus*. Phk-3 is also expressed in macrophage-like S2 cells, and its expression is enhanced when treated with LPS, suggesting that Pherokines might participate in the immune reaction [47].

There are other mechanisms for the symbiont-induced overexpression of OBPs to enhance the host immune capacity. In the tsetse fly *Glossina morsitans morsitans*, the obligate symbiont *Wigglesworthia* upregulates *obp6* expression in the gut of intrauterine tsetse larvae. This process is essential and sufficient to trigger the systemic expression of the hematopoietic RUNX transcription factor *lozenge*, leading to the subsequent generation of crystal cells (a type of hemocyte) that could activate a melanotic immune reaction in adult tsetse [38].

Overall, multiple functions of pathogen-induced OBP overexpression in host chemoreception and immunity will elicit host immune priming, where earlier experience with a low dosage of pathogen infection affords defense against subsequent infection with the same pathogen [48]. A counter-intuitive result is found in the migratory locust *Locusta migratoria*. The pathogenic fungus *Metarhizium anisopliae* generates phenylethyl alcohol (PEA), which induces behavioral repellence in locusts. *LmOBP11* participates in the insect detection of PEA and aversion of food contaminated with PEA, but the upregulation of *LmOBP11* expression upon *M. anisopliae* infection has a negative impact on insect immune reactions. Furthermore, the expression of an extracellular serine protease that is involved in generating the *Drosophila* Toll ligand Spätzle is markedly upregulated in locusts co-treated with *LmOBP11* dsRNA and *M*. *anisopliae* infection, suggesting that *LmOBP11* acts as a negative regulator modulating immune-protease-related functions [39]. This finding manifests a communication between olfaction and immunity, suggesting that the manipulation of host OBPs as a potential target is exploited by fungal pathogens to suppress immune activation and thus facilitate their colonization in the host. Future efforts should focus on identifying whether the regulatory function of OBPs is exclusive to locusts or also exists in other insect pests or vectors of infectious disease.

Infection with certain microbes could also suppress host OBP expression. A high load of DWV or parasitism by a microsporidian parasite *Nosema ceranae* induces a downregulation of OBP expression in *A*. *mellifera*, which could then lead to a reduction in host olfactory sensitivity [20,33]. One OBP domain protein in the mosquito *Anopheles gambiae*, OBPd-1, is downregulated by the *Salmonella typhimurium* infection [49]. The inoculation of female *Aedes aegypti* with a double subgenomic *Sindbis virus* leads to a significant reduction in *AaegObp1* and *AaegObp2* mRNA levels in olfactory tissues compared with controls, suggesting that this virus expression system can be utilized to effectively suppress OBP expression [30].

### 4.2. Regulating Host OR/GR Expression

Microbial symbionts can alter insect olfactory perception by manipulating the expression of ORs. Infection with *Wolbachia* increases the sensitivity of *D. simulans* to food odors by enhancing the host transcript levels of the odorant receptor co-receptor gene *or83b* [25]. *Spodoptera exigua multiple nucleopolyhedrovirus* (SeMNPV) is an entomopathogenic double-stranded DNA virus that mainly infects the beet armyworm *Spodoptera exigua*. OR35 is upregulated in larvae infected with SeMNPV, which renders *S*. *exigua* to recognize a broader spectrum of odor molecules with diverse chemical structures [41]. In the case of plant viruses, infection with *Rice stripe virus* significantly upregulates olfactory receptor co-receptor transcripts in the small brown planthopper *Laodelphax striatellus*, resulting in enhanced olfactory sensitivities and improved seeking abilities to the odor of rice plants compared with healthy insects [50].

The gut microbiota may modulate host taste preferences by regulating the expression of GRs. While associations between gut microbiota and host gustatory functions have not been systematically illustrated in insects, a possible mechanism of receptor manipulation by gut bacteria has been found in the studies of germ-free murine. For example, germ-free mice possess a larger number of sweet receptors and higher preferences for sweet compared with normal mice, which may subsequently influence the structure of the gut microbial community and increase the risk of metabolic disorders [51,52]. This role of gut microbes extends beyond sweet tastants. In a similar study, germ-free mice exhibit a higher preference for intralipid emulsion, which is linked to alterations in lingual and proximal gut fatty acid receptors [53]. These studies demonstrate that the missing of microbes can result in an altered receptor expression and potential changes in gustatory perception. However, it is not revealed what mechanisms or systems microbes exploit to sustain their influence on gustation [54].

### 4.3. Regulating Host ODE Expression

The metabolism of signal molecules by ODEs is essential to maintaining the sensitivity and specificity of chemoreception in different insects, and several specific esterases, cytochrome P450s (CYP450s), glutathione S-transferases (GSTs), and UDP-glycosyltransferases (UGTs), are involved in this process [55]. At present, the manipulation of insect ODEs by microbial symbionts is seldom reported. In mammals, CYPs, GSTs, and UGTs are highly expressed in the olfactory epithelium and presumed to participate in odorant transformation, degradation, and/or olfactory signal termination [56]. Expression of the genes encoding the main CYP450, GST, and UGT isozymes is downregulated in the olfactory epithelium of germ-free mice [57]. Considering that these enzymes may be in charge of odorant decomposition, such a reduction could potentially result in prolonged responses to odorant stimulation.

### 4.4. Regulating Host Neurotransmission

It has been proposed that elevated amounts of microbiota-produced chemical messengers may be a crucial intermediate for crosstalk between bacteria and host neurons [58]. Several gut bacterial strains, such as *Bacillus subtilis*, *Escherichia coli*, and *Lactobacillus plantarum*, are known to generate neurotransmitters such as dopamine, acetylcholine, serotonin, noradrenaline, and gamma-aminobutyric acid (GABA) [59]. GABA produced by gut microbiota impacts the host homeostatic production of GABA by the local neurons that modulate olfactory receptor neurons (ORNs), and/or the expression of GABA receptors on the ORN terminals. Any disruption in this homeostasis may impact information processing in the host olfactory circuit, subsequently affecting the behavioral responses of the host to odorants [24]. Furthermore, in the nematode *Caenorhabditis elegans*, the neuromodulator tyramine generated by commensal *Providencia* bacteria is probably transformed to octopamine by the tyramine β-hydroxylase enzyme of the host. Octopamine subsequently targets the OCTR-1 octopamine receptor on ASH nociceptive neurons to regulate a repellent olfactory response [34].

Microbial symbionts can also regulate host chemical messenger biosynthesis. Dopaminergic signaling plays a pivotal role in regulating insect activity. Two essential genes in the dopamine biosynthesis pathway, *Pale* and *Ddc*, are upregulated in *Wolbachia*-infected *D. melanogaster* [37]. Similarly, *Wolbachia*-infected *A*. *aegypti* has higher dopamine levels in their heads [31]. Dopamine and serotonin that suppress the sensory sensitivity of honeybees are downregulated in the brain of bacteria-colonized individuals, which promotes host sucrose sensitivity [43]. This phenomenon has also been discovered in mammals. The amount of dopamine released in infected glia cells is improved threefold upon infection with *Toxoplasma gondii* compared with uninfected cells and, consequently, the aversion of mice to cat odor is changed into attraction [60].

The symbiotic system of *D. melanogaster* and *Erwinia carotovora carotovora 15* (*Ecc15*) is a typical example to explain how microbial symbionts regulate the host nervous system [35]. Flies without gut bacterial infection can eat more food containing *Ecc15*. However, flies infected with *Ecc15* show significant aversion to food containing *Ecc15*, indicating that *Ecc15* infection increases host olfactory discrimination. It is further found that *Ecc15* infection can regulate fly olfaction via the metabolic reprogramming of ensheathing glia of the antennal lobe. Gut-derived inflammatory cytokines induced by *Ecc15* modulate glia/neuron metabolic coupling in the fly brain, which increases lactate and lipid transport between olfactory neurons and glia and consequently results in lipid droplets accumulation and the upregulation of mitochondrial β-oxidation in glia. Increased oxidative stress from β-oxidation results in the age-related dysfunction of cholinergic projection neurons within the olfactory circuit, thus inducing an adaptive temporary halt of olfactory discrimination upon gut infection. Such alterations in the olfactory system help to enhance the avoidance of flies to bacteria-laced food and promote fly survival [61].

Microbial symbionts also affect olfactory sensitivity via interference with the host olfactory memory. *Wolbachia* infection shortens the memory duration of the wasp *Trichogramma brassicae* compared with uninfected individuals and thus stimulates the dispersal of the endosymbiont by manipulating hosts to forget the information of prior environments [42]. The stimulation of the immune system of honeybees with non-pathogenic elicitors, such as LPSs, reduces learning abilities in *A*. *mellifera*, which are less able to associate an odor with a reward [62]. *N*. *ceranae* could modulate the immune system of honeybees by enhancing oxidative responses or altering the expression of antimicrobial peptides [63]. It is thus possible that the observed impairment of olfactory learning behavior in bumblebees exposed to *N*. *ceranae* is due to an activation of the immune reaction [64]. Moreover, *N*. *ceranae* also disturbs the serotonin metabolism by, for instance, suppressing the expression of the dopa decarboxylase-encoding gene [65], a biogenic amine that plays an important function in the process of memory formation in *Drosophila* [66].

Conversely, sterile *Drosophila* displays a moderate decline in memory performance during olfactory appetitive conditioning compared with normal flies [36]. The mushroom body in *D. melanogaster* is a neural circuit that plays a crucial role in the formation and storage of memories [67]. Numerous researchers have surveyed the influence of *Ecc* on the olfactory nervous system of *Drosophila*, and it is assumed that the ingestion of *Ecc* strengthens synaptic connections between the neurons that transport unconditioned stimulus information and the mushroom body Kenyon cells, thereby enhancing olfactory-conditioned stimulus information in insects [68]. Using laboratory-generated gnotobiotic honeybees, it is found that gut *Lactobacillus* is required for olfactory learning and memory abilities. Aromatic amino acid aminotransferase (ArAT) converts tryptophan to serotonin, kynurenine (Kyn), and indolic compounds, which exert a profound influence on gut–brain interaction. ArAT produced by gut *Lactobacillus* strains could upregulate tryptophan metabolism, thus improving the learning and memory performance of honeybees [32].

## 5. Evolutionary Significance of Symbiont-Mediated Chemoreception

The manipulation of insect chemosensory behavior by microbial symbionts is conducive to their proliferation and dispersal. If such an association is beneficial to both partners, it can promote the co-evolution of the symbiotic relationship. Leafcutter ants are polyphagous herbivores that possess the ability to avoid plants containing chemicals detrimental to their symbiotic fungi. Therefore, the preference of leafcutter ants for plants is partly driven by the demand for fungi, which protects mutually symbiotic fungi from harmful compounds [69]. *Drosophila paulistorum* mating with the ‘wrong’ partner (belonging to another incompatible semi-species) is harmful for both the symbiont and the host (obligate intracellular/bidirectional cytoplasmic incompatibility plus hybrid sterility). Therefore, this symbiotic relationship has developed delicate modalities to recognize the infection type of the latent mate before mating by active mate avoidance [70].

Infection with parasites or pathogens adversely affects the host, and insects can change their feeding habits by enhancing their chemical sensing to meet the demand of their own survival and reproduction. Infection with lethal parasites changes the taste of specific phytochemicals, such as pyrrolizidine alkaloids and iridoid glycosides, in *G*. *geneura* larvae. Considering that these compounds are toxic to parasites and present in plants eaten by caterpillars, their altered taste may stimulate parasitized individuals to ingest more plants that offer a biochemical defense against parasites [28]. Furthermore, antennal and related olfactory proteins are candidates for mediating the host detection of pathogens and immune reactions to pathogens, which may be conducive to the subsequent behavioral and/or immune reactions of the host to the infection challenge [44]. This may be a more precise evolutionary pathway that allows the hosts to protect themselves from pathogens.

The suppression of host chemosensitivity induced by parasite or pathogen infection will affect host aggregation and dispersal behavior, which facilitates population segregation. Infection with the microsporidian parasite *Paranosema locustae* suppresses the aggregation of solitary *Locusta migratoria manilensis* and causes gregarious individuals to shift back to solitary behavior. Healthy locusts exposed to olfactory stimuli from parasite-infected conspecifics exhibit a significant reduction in serotonin levels, leading to decreased gregariousness [71]. This transformation is not conducive to the spread of parasites but is conducive to the survival and development of locust populations [72]. Infection with the fungal pathogen *Erynia neoaphidis* decreases the sensitivity of the pea aphid *Acyrthosiphon pisum* to alarm pheromone, and the percentage of nonresponding aphids increases as the disease progresses [73]. Although such behavior benefits the intrapopulation transmission of the pathogen, it suppresses the dispersal of infected aphids and transmission of this pathogen to other healthy populations. If the host could survive the pathogen infection, they will contribute to population differentiation.

## 6. Summary and Prospect

Symbiotic associations between insects and microbial symbionts are universal in nature. Symbiosis offers a chance for microbial symbionts to live together through a mutual or one-way benefit with insects. Insect chemoreception system perceives volatiles, and microbial symbionts can cause alterations occurring at any point in the process from recognizing the signal to transmitting it to the brain to elicit a response. Symbionts not only impact insect chemoreception through interference with the expression of host OBPs/CSPs, ORs/GRs, and ODEs but also fine-tune host chemosensory neurotransmission, thus regulating insect behavior. Microbial symbiont-mediated chemoreception facilitates the spread of symbionts but could be beneficial or detrimental to the hosts, which provides an impetus for the co-evolution of symbiotic systems.

Although insect-symbiont interactions have always been a research hotspot in the insect ecological field, the molecular mechanisms by which gut microbes modulate host physiology, however, remain largely unknown [74]. Future research is necessary to reveal the exchange of material and information between microbial symbionts and hosts, such as the mechanisms by which symbionts affect brain functions and the central nervous system [9], and whether the GABA produced by gut microbiota directly influences olfactory neurons or there are sensory afferents that detect GABA in the gut, which subsequently activates the CNS to regulate olfactory functions [24]. The associations between symbionts and their hosts can be unraveled with a variety of experimental tools, such as bio-chemical and -omics approaches (metagenomics, transcriptomics, and metabolomics). The integration of these tools in future studies will improve our comprehension of the evolution of chemoreception and chemical ecology in insects, providing insight into species interactions, speciation, conservation, pest management, and environmental restoration [75,76,77].

Manipulating insect physiology by inoculating them with microbes is a potential method to reduce the application of insecticides in pest management. The double subgenomic *Sindbis virus* expression system can suppress OBP gene expression in the olfactory organs of mosquitoes [30], which provides a crucial step towards disrupting the host-seeking behavior of mosquitoes and preventing disease transmission. However, further functional and genetic studies are needed to fully evaluate the risk of the release of genetically modified organisms and their potential in pest control. Moreover, olfactory-related genes with an altered expression after pathogen infection could be screened as targets for RNAi, which could be used together with pathogen-based formulations to improve the pest control efficacy. For example, the feeding behavior of the rice planthopper *Sogatella furcifera* to *Southern rice black*-*streaked dwarf virus*-infected rice plants switches from preference to non-preference after *M*. *anisopliae* infection. Interestingly, the OR23 and OR43 are downregulated after pathogenic fungal infection and could be used as targets to develop strategies (e.g., RNAi to target OR) to suppress the capability of *S*. *furcifera* to transmit this virus [78].

## Data Availability

The data presented in this study are available on request from the corresponding author.
